# The genera *Deuterixys* Mason, 1981 and *Wilkinsonellus* Mason, 1981 (Hymenoptera, Braconidae, Microgastrinae) from China, with description of two new species

**DOI:** 10.3897/zookeys.120.891

**Published:** 2011-07-25

**Authors:** Jie Zeng, Jun-Hua He, Xue-Xin Chen

**Affiliations:** Institute of Insect Sciences, Zhejiang University, 268 Kaixuan Road, Hangzhou 310029, China

**Keywords:** Hymenoptera, Braconidae, Microgastrinae, Cotesiini, *Deuterixys*, *Wilkinsonellus*, new species, key, China

## Abstract

The genus *Deuterixys* Mason, 1981 of the tribe Cotesiini (Hymenopteran, Braconidae, Microgastrinae) is recorded from China for the first time. Two new species, *Deuterixys bifossalis* Zeng & Chen, **sp. n.** and *Deuterixys curticalcar* Zeng & Chen, **sp. n.**, are described and illustrated, and a key to the Old World species of *Deuterixys* is given. In addition, *Wilkinsonellus paramplus* Long & van Achterberg, 2003 is recorded from China for the first time and illustrated.

## Introduction

The tribe Cotesiini (Braconidae, Microgastrinae) was established by Mason (1981) with most members parasitizing on Macrolepidoptera. A few species of this tribe have been used in biological control of lepidopteran pests. The genera *Deuterixys* and *Wilkinsonellus* are two small genera of this tribe, and both characterized by a longitudinal median groove at least at basal half of the first tergite and without an areolet of fore wing.

The genus *Deuterixys* proposed by Mason (1981) includes four Old World species of Nixon’s (1965, 1976) *carbonarius* group of *Apanteles* Förster. This genus includes 14 described species widespread in the world except in the Afrotropical region, of which seven occur in the Old World and seven in the New World (Nixon 1965; Tobias 1971, 1976; Papp 1983b, 1990; Whitfield 1985; Austin and Dangerfield 1992; Marczak and Buszko 1994; Yu et al. 2005). Most species of the known hosts of this genus are leaf miners, including the genera *Bucculatrix* and *Leucoptera* of the family Lyonetiidae and *Stigmella* of the family Nepticulidae (Lyle 1925; Wilkinson 1936, 1940; Telenga 1955; Nixon 1965; Tobias 1976, 1986; Papp 1983a; Whitfield 1985; Marczak and Buszko 1994; Gates et al. 2002), which may cause damage in forests. Therefore, species of *Deuterixys* may play an important role in controlling those forest pests. Recently two new species of this genus are found in China among specimens of Parasitic Hymenoptera Collection of Zhejiang University (ZJUH) and described in this paper. They represent the first record of the genus *Deuterixys* Mason for China.

The genus *Wilkinsonellus* was proposed by Mason (1981) to include four Old World species of Nixon’s (1965) *henicopus* and *daira* group of *Apanteles* Förster, including five described species occurring in Oriental region, four in the Australasian region and one in the Afrotropical region (De Saeger 1944; Nixon 1965; Austin and Dangerfield 1992; Chou 1999; Long and van Achterberg 2003; Chen and Song 2004; Ahmad et al. 2005; Yu et al. 2005). One species, *Wilkinsonellus iphitus* (Nixon 1965) was previously recorded from Hainan and Taiwan of China (Chou 1999; Chen and Song 2004). Here we report another species of this genus, *Wilkinsonellus paramplus* Long & van Achterberg, 2003, from China for the first time.

## Material and methods

Specimens studied are deposited in the Parasitic Hymenoptera Collection of Zhejiang University, Hangzhou, China (ZJUH). Descriptions and measurements were made under a stereomicroscope (Zeiss Stemi 2000-C). All figures were made by a camera (Q-Imaging, Micropublisher, 3.3 RTV) attached to a stereomicroscope (Leica MZ APO, Germany) and Auto-Montage Pro version 5.0 software.

Terminology and measurement follows Nixon (1965) and Mason (1981), vein terminology follows the modified Comstock-Needham system (van Achterberg 1979). Abbreviations used in this paper are as follows: POL = postocellar line, OOL = ocular-ocellar line, OD = ccellar diameter; TI = the first tergite of metasoma, TII = the second tergite of metasoma, TIII = the third tergite of metasoma; L = length, W = width.

## Taxonomy

### Genus Deuterixys

Mason, 1981

http://species-id.net/wiki/Deuterixys

Deuterixys Mason 1981, 115: 123; Whitfeild 1985, 61(1): 60; Marsh et al. 1987, 13: 31; Whitfield and Wagner 1991, 25: 737; Austin and Dangerfield 1992, 6(1): 23.

#### Type species:

*Microgaster carbonarius* Wesmael, 1835. Designated by Mason 1981.

#### Diagonosis.

Areolet of fore wing absent; propodeum polished and bearing a strong long medial carina; TI of metasoma with medio-basal longitudinal groove; TII and III broad, rectangular, and noticeably constricted or abruptly widened at the suture between them; ovipositor sheaths short, decurved and subexerted.

#### Key to the Old World species of the genus *Deuterixys* Mason, 1981

**Table:** 

1	TII+III enlarged to form a coarsely rugose carapace that completely hides the more apical segments; posterior margin of this carapace finely crenulate laterally. [Mesoscutum shiny, closely, rather strongly punctate for the size of the insect; hind coxa and underside of metasoma bright yellow; propodeum polished and with a strong medial keel; length: 1.8 mm]	*Deuterixys patro* Nixon, 1965
–	TII+III not thus enlarged, notched at the position of second suture but its posterior margin is membranous and smooth, the more apical segments exposed	2
2	TII less transverse, at most 1.7–1.8 times wider behind than long medially; legs yellow, hind tibia and tarsus more or less infuscate	3
–	TII more transverse, twice wider behind than long medially; legs dark brown, blackish to black	4
3	Vein 2-CU1 of fore wing twice as long as vein 1-CU1; TIII rugose to rugulose; preapical segment of antenna short, hardly twice as long as wide	*Deuterixys condarensis* (Tobias, 1960)
–	Vein 2-CU1 of fore wing as long as vein 1-CU1; TIII smooth and shiny; preapical segment of antenna long, more than twice as long as wide	*Deuterixys bifossalis* Zeng & Chen, sp. n.
4	TIII densely rugulose or subrugulose, dull; TII with rugosity almost similar to that of TI; TI parallel- or indistinctly subparallel-sided; propodeum medially with more or less transverse rugulosities along medial longitudinal keel, otherwise propodeum smooth	5
–	TIII chagreened or almost smooth, shiny; TI converging apically; propodeum smooth, at most with a few and very short rugulae along hind carina and above lunule	6
5	TI subrectangular, 1.2–1.3 times as long as wide; TII almost as long as TIII; inner hind tibial spurs much longer than half length of hind basitarsus	*Deuterixys carbonaria* (Wesmael, 1837)
–	TI more than 1.5 times longer than wide; TII distinctly longer than TIII; inner hind tibial spurs shorter than half length of hind basitarsus	*Deuterixys curticalcar* Zeng & Chen, sp. n.
6	Vein 1-R1 of fore wing as long as or slightly shorter than pterostigma. [TI posteriorly weakly to moderately converging, its basal width at most 1/3 greater than its apical width. Pterostigma less wide, 2.7–2.9 (-3.0) times longer than wide. First tergite always entirely black. Tegula brownish black to brownish yellow. Middle and hind femora black(ish)]	*Deuterixys rimulosa* (Niezabitowski, 1910)
–	Vein 1-R1 slightly longer than pterostigma	7
7	TI wedge-shaped, 1.6–2 times longer than wide at base; TII shorter than TIII, somewhat less transverse, twice wider behind than long medially; pterostigma 2.3–2.4 times longer than wide	*Deuterixys plugarui* (Tobias, 1975)
–	T1 widest about 2/3 distance from its base, anterior third and posteriorly with weakly converging sides, about 1.5 times longer than wide; TII slightly longer than TIII, somewhat more transverse, 2.2 times wider behind than long medially; pterostigma about 3 times longer than wide	*Deuterixys anica* Austin & Dangerfield, 1992

### Deuterixys bifossalis

Zeng & Chen sp. n.

urn:lsid:zoobank.org:act:B29EDB9A-C1FD-4FF6-BD77-94DB7D91236C

http://species-id.net/wiki/Deuterixys_bifossalis

Figs 1–7

#### Description.

##### Female.

Body length 3.68 mm, fore wing length 3.20 mm.

Head. In frontal view antennal sockets just above middle level of eyes, 1.6 times as wide as long and 1.1 times as that of mesoscutum. Ocelli large and in a equilateral triangle, POL: OD: OOL=3.6:4.0:2.7. Frons and vertex smooth and shiny, scattered with short fine setae; vertex sharply narrowed behind eyes, area behind ocellar area sharply oblique, smooth and shiny, without setae; temple and gena feebly punctate and shiny, with dense setae. Face and clypeus shiny but feebly rugulose-punctate, with dense short fine setae; width of face 0.5 times height of eye and clypeus combined (16.3:31.2); inner margins of eyes adjacent to face parallel-sided; eyes very large, 1.5 times as high as wide (31:21), temple behind eyes very short. Tentorial pits large, distance between tentorial pits 5 times distance from pit to eye margin; malar space very short, 0.1 times as long as eye height; apical segment of labial palp longer than the two preceding segments, respectively. Antenna longer than body; flagellomeres with placodes arranged regularly in 2 ranks; the third flagellum slightly longer than the fourth flagellum; apical segment as long as preapical one; preapical segment 2.3 times as long as wide. Flagellomere proportions: 2 L/W=2.92, 8 L/W=2.40, 14 L/W=2.50; L 2/14=1.40; W 2/14=1.20.

Mesosoma. Mesoscutum densely and evenly punctate and setose; notauli not impressed, but indicated by a band of shallow and dense duller sculpture. Disc of scutellum also densely punctate, its rugose tip interrupting the posterior, polished band of scutellum, with dense short setae all over; scutellar sulcus deep with a few strong carina and 1.2 times as long as scutellum (15.5:12.8). Propodeum highly polished, virtually without sculpture except for a strong medial longitudinal carina and weak transverse ridges in immediate vicinity of longitudinal carina and lateral margin, with strong rugae distal to spiracle. Epicnemial furrow distinct, area before it raised above rest of mesopleuron; precoxal sulcus short, shallow, only indicated by few punctures anteriorly. Mesosternum with dense setigerous punctures. Lateral metanotum mostly smooth and shiny, with longitudinal striae posteriorly and below spiracle.

Wings. Forewing without areolet, radial vein r arising from distal third of pterostigma; veins r and 2-SR meeting at a 165~170 degree angle; r:2-SR: length of pterostigma = 13:10:31; vein 1-R1 1.3 times as long as pterostigma, pterostigma 2.5 times as long as wide. 1-CU1:2-CU1:m-cu=11:11:8. Hind wing narrow.

Legs. Hind coxa shiny, feebly punctate, scattered with short setae. Hind tibia gradually swollen apically and about 0.9 times as long as hind tarsa (54.5:63.9); inner hind tibial spurs about 0.9 times as long as hind basitarus (22.0:25.8); forth tarsal segment slightly shorter than fifth tarsal segment (7.8:8.4). Hind tibia with few spines on outer side, rather fine and not dense enough to give the tibia a markedly prickly appearance.

Metasoma. TI dilated medially, the greatest medial width 1.5 times its apical width, with strong medial longitudinal groove almost reaching to posterior margin, 1.5 times as long as its greatest width and 2.1 times as long as TII, medial groove with a shallow transverse carina. TII+III slightly constricted at extreme apex of second suture. TII subtrapezoid, 0.6 times as long as its basal width and 0.9 times as long as TIII, with a pair of strong rugulose-marked longitudinal grooves delimiting a median field that slightly narrowed posteriorly. TIII rectangular, smooth and shiny, with anterior margin arched medially, median field not indicated. Tergites posterior to TIII more membranous, shiny. All tergites scattered with setae. Ovipositor sheath shorter, 0.7 times as long as hind basitarsus. Hypopygium strongly and evenly sclerotized, blunt but not truncated apically, sparsely clothed with short setae.

Colour. Body mostly yellow; head evenly yellow except for black ocellar area; mesosoma brown except for yellow mesoscutum and scutellar disc; mesopleuron yellow except epicnemium. Antenna evenly yellowish brown, scape brownish dorsally, pedicel yellow; palpi and tegula whitish yellow. Legs whitish yellow basally, slightly darkened toward apex, claws brown, extreme apex of hind femur and tibia and extreme base of hind tibia brownish. Sternites of metasoma whitish yellow; TI light yellow, the other tergites brown and gradually paler apically. Wings hyaline, very slightly infuscate; veins brown but veins 1-R1, A and M+Cu grey, pterostigma brown.

##### Male.

Unknown.

#### Host.

Unknown for this species, but all previous host records from Europe and North America indicate that *Deuterixys* spp. are parasites of *Bucculatrix* spp. (Bucculatricidae) (Nixon 1965, Mason 1981, Whitfield 1985).

#### Materials examined.

Holotype: ♀, Mt. Qingliangfeng (118°52'E, 30°04'N; 119°12'E, 30°31'N), Linan, Zhejiang, 2005. VIII. 9, leg. Min Shi, No. 200607234. Paratype: 1♀, Mt. Yinggeling (109°31'E, N19°04'N), Hain0061n, 2007. V. 24–25, leg. Jingxian Liu, No. 200702641.

#### Etymology.

The specific name “bifossalis” derives from the Latin prefixion “bi-” and adjective “fossalis”, referring to both distinct lateral grooves enclosing a median field of the second tergite.

#### Distribution.

China (Zhejiang, Hainan).

#### Remark.

This species is similar to the Palaearctic species *Deuterixys rimulosa* (Niezabitowski, 1910), but can be distinguished from the latter by having TI not transverse (more transverse in *Deuterixys rimulosa*), and vein 1-R1 of fore wing longer than pterostigma (the latter subequal to or slightly shorter). It also differs from the other Oriental species *Deuterixys patro* (Nixon, 1965) by TII and TIII not forming a carapace and having exposed the following tergites (the latter TII and TIII enlarged to form a carapace and following tergites retracted).

**Figures 1–7.**
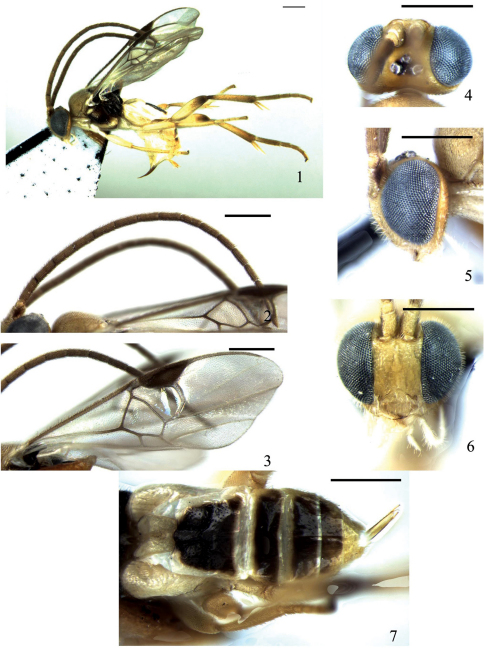
*Deuterixys bifossalis* Zeng & Chen, sp. n. **1** habitus, lateral view **2** antennae **3** fore wing **4** head, dorsal view **5** head, lateral view **6** head, frontal view **7** gaster, dorsal view. Scale line = 0.5 mm.

### Deuterixys curticalcar

Zeng & Chen sp. n.

urn:lsid:zoobank.org:act:34B734EE-BBE6-44D0-AA54-8275B151633F

http://species-id.net/wiki/Deuterixys_curticalcar

Figs 8–14

#### Description.

##### Female.

Body length 2.04 mm, fore wing length 2.24 mm.

Head. In frontal view antennal sockets distinctly above middle level of eyes, head 1.6 times as wide as long and 1.1 times as that of mesoscutum. Ocelli small and in a high triangle, POL: OD: OOL=4.0:2.4:6.2. Frons and vertex smooth and shiny, covered with dense short fine setae; vertex sharply narrowed behind eyes, area behind ocellar area sharply oblique, smooth and shiny, without setae; temple and gena feebly striate and shiny, with dense setae; face shiny and discretely but distinctly punctate, 0.9 times as wide as height of eye and clypeus combined, with dense setae; inner margins of eyes adjacent to face slightly converging ventrally; eye moderate size, 1.7 times as high as wide (15.6:9.0), temple behind eyes subequal in length to width of eye. Clypeus feebly rugulose, with dense short fine setae; tentorial pits small, distance between tentorial pits twice distance from pit to eye margin (5.7:2.8); malar space short, 0.2 times as long as eye height; apical segment of labial palp longer than the preceding segment but shorter than the next preceding segment. Antenna longer than body; flagellomeres with placodes arranged regularly in 2 ranks except the apical 6 segments; the third flagellomere subequal to the forth flagellomere in length; apical segment slightly longer than preapical one. Flagellomere proportions: 2 L/W=4.10, 8 L/W=4.05, 14 L/W=1.88; L 2/14=2.00; W 2/14=0.92.

Mesosoma. Mesoscutum densely and evenly punctate, with normal setae; notauli absent. Disc of scutellum as densely punctate as mesoscutum, with normal setae; the posterior, polished band of scutellum continuous, not interrupted medially; scutellar sulcus shallow with a few weak carinae and as long as scutellum. Propodeum highly polished, virtually without sculpture except for a strong medial longitudinal carina and very short transverse ridges in immediate vicinity of longitudinal carina and lateral margin, with strong rugae around spiracle. Epicnemial furrow indistinct, area before it slightly raised above rest of mesopleuron; precoxal suture indistinct, only indicated by a large shallow depression. Mesosternum with dense fine setigerous punctures. Laterally metanotum mostly smooth and shiny.

Wings. Forewing without areolet, radial vein r arising from middle of pterostigma; veins r and 2-SR meeting at an angle of almost 170 degree; r:2-SR:length of pterostigma = 7.5:6.5:23.0; vein 1-R1 1.1 times as long as pterostigma, pterostigma 2.6 times as long as wide. 1-CU1:2-CU1: m-cu=6.5:7.0:7.3. Hind wing narrow.

Legs. Hind coxa shiny, highly polished, scattered with short setae on anterior 2/3. Hind tibia gradually swollen apically and 0.9 times as long as hind tarsus (33.0:38.8); inner hind tibial spurs 0.4 times as long as hind basitarsus (6.0:15.5); forth tarsal segment much shorter than fifth tarsal segment (4.5:7.0). Hind tibia without trace of spines on outer side.

Metasoma. TI parallel-sided, with a very feebly rugose triangular area and a strong and smooth medial longitudinal groove on anterior 2/5, densely rugulose and turns over on posterior 3/5, 1.6 times as long as its greatest width and 1.5 times as long as tergite II. TII+III constricted at the level of the crenulate second suture. TII rectangular, densely and strongly rugose, with strong longitudinal striae posteriorly, without trace of median field, 0.6 times as long as its basal width and 1.7 times as long as tergite III. TIII transverse, broadened posteriorly, longitudinally aciculate-rugulose and densely and strongly rugulose. Tergites posterior to TIII more membranous, shiny; all tergites with scattered short fine setae. Ovipositor sheath very short, only 0.4 times as long as hind basitarsus, curved downwards. Hypopygium short, strongly and evenly sclerotized, truncated apically, sparsely clothed with long setae.

Colour. Body mostly black; metasoma brownish to light brown except black TI to TIII. Antenna almost brown, basal third yellowish ventrally. Mouthparts yellow with brown margin, palpi white. Tegula brown. Legs yellow, somewhat whitish; fore and middle coxae brownish laterally, all claws brown; hind coxa and tarsus brown; hind tibia brownish and darkened apically, its apical third strongly darkened. Wings hyaline; veins grey except brown submarginal vein, pterostigma grey and laterally brown.

Variation. Vein 1-SR 1.1–1.4 times as long as pterostigma, pterostigma 2.6–3 times as long as wide. Veins and pterostigma light brown, more or less transparent. Antenna, mouthparts and tegula sometimes darkened.

##### Male.

Unknown.

#### Host.

Unknown for this species but all previous host records from Europe and North America indicate that *Deuterixys* spp. are parasites of *Bucculatrix* spp. (Bucculatricidae) (Nixon 1965, Mason 1981, Whitfield 1985).

#### Materials examined.

Holotype: ♀, main peak of Mt. Huping (110°45E, 30°02'N ~ 110°55'E, 30°07'N), Shimen, Hunan, 2009. VII. 12, legs. Zeng Jie, No. 200900720. Paratype: 2♀♀, Main peak of Mt. Huping (110°45'E, 30°02'N ~ 110°55'E, 30°07'N), Shimen, Hunan, 2009. VII. 12, legs. Zeng Jie, No. 200900716, 200900745; 1♀, Mt. Huping (110°45'E, 30°02'N ~ 110°55'E, 30°07'N), Sanhecun, Shimen, Hunan, 2009. VII. 13, legs. Zeng Jie, No. 200901663; 2♀♀, Mt. Huping (110°45'E, 30°02'N ~ 110°55'E, 30°07'N), Sanhecun, Shimen, Hunan, 2009. VII. 11, legs. Tang Pu, No. 200901072, 200901079; 2♀♀, Mt. Huping (110°45'E, 30°02'N ~ 110°55'E, 30°07'N), Sanhecun, Shimen, Hunan, 2009. VII. 11, legs. Ma Li, No. 200901910, 200901952; 1♀, Mt. Huping (110°45'E, 30°02'N ~ 110°55'E, 30°07'N), Sanhecun, Shimen, Hunan, 2009. VII. 13, legs. Ma Li, No. 200901011; 1♀, Mt. Jingang(114°06'E, 26°31'N ~ 114°10'E, 26°34'N), Jiangxi, 2007. VIII. 13, legs. He Junhua, No. 200704967; 1♀, Jietou (98°37'E, 25°22'N ~ 98°39'E, 25°27'N), Tengchong, Yunnan, 2006. VII. 11–12, legs. Zeng Jie, No. 200801766; 1♀, Nankang (98°46'E, 24°48'N ~ 98°47'E, 24°49'N), Lujiangba, Baoshan, Yunnan, 2009. V. 9, legs. Zeng Jie, No. 200904232; 1♀, Mt. Leigong (118°03'E, 26°21'N ~ 118°15'E, 26°25'N), Xiaodanjiang, Guizhou, 2005. VI. 4, legs. Zhang Hongying, No. 200606086; 1♀, Kuankuoshui Natural Reserve(107°24'E, 30°37'N ~ 107°24'E, 30°37'N), Guizhou, 2010. VI. 5, legs. Zeng Jie, No. 201004665; 1♀, Datianding, Mt. Dawuling (111°11'E, 22°16'N ~ 111°13'E, 22°18'N), Guangdong, 2001. X. 3, legs. Xu Zaifu, No. 20020629; 1♀, Mt. Chebaling (114°14'E, 24°43'N ~ 114°16'E, 24°44'N), Shixing, Guangdong, 2003. VIII. 21, legs. Xu Zaifu, No. 20053046; 2♀♀, Baotianman (111°55'E, 33°29'N ~ 111°58'E, 33°32'N), Neixiang, Henan, 1998. VII. 14, legs. Chen Xuexin, No. 988733, 988741; 1♀, Hongxia tree farm, Mt. Liupan (106°13'E, 35°43'N ~ 106°17'E, 35°45'N), Jingyuan, Ningxia, 2008. VI. 1, legs. Liu Jingxian, No. 200905594; 1♀, Guamagou, Mt. Liupan (106°19'E, 35°46'N ~ 106°21'E, 35°47'N), Pengyang, Ningxia, 2008. VI. 9–10, legs. Liu Jingxian, No. 200904351; 1♀, Yehegu, Xixia tree farm, Mt. Liupan (106°13'E, 35°29'N ~ 106°17'E, 35°31'N), Jingyuan, Ningxia, 2008. VI. 11–12, legs. Liu Jingxian, No. 200905857.

#### Etymology.

The specific name “curticalcar” derives from the Latin prefixion “curti-” and noun “calcar”, referring to the short hind tibial spurs.

#### Distribution.

China (Jiangxi, Henan, Hunan, Guangdong, Guizhou, Yunnan, Ningxia).

#### Remark.

This species is similar to the Palaearctic species *Deuterixys carbonaria* (Wesmael, 1837), but can be distinguished from the latter by having TI long, more than 1.5 times as long as wide (TI subrectangular, 1.2–1.3 times as long as wide in *Deuterixys carbonaria*); TII much longer than TIII (TII almost as long as TIII); and inner hind tibial spurs shorter than 0.5 times hind basitarsus (much more than 0.5 times hind basitarsus).

**Figures 8–14.**
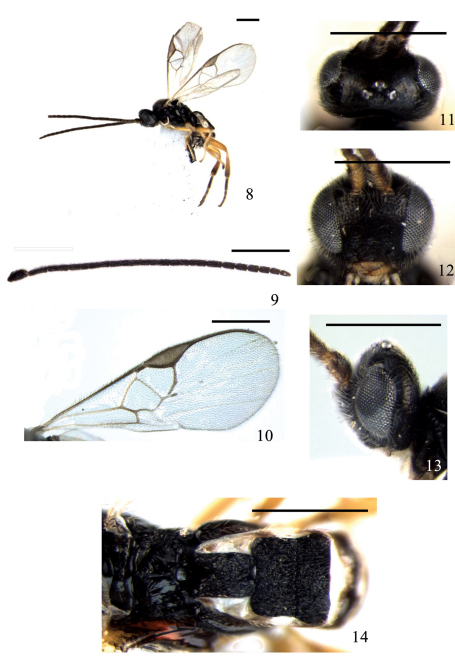
*Deuterixys curticalcar* Zeng & Chen, sp. n. **8** habitus, lateral view **9** antennae **10** fore wing **11** head, dorsal view **12** head, frontal view **13** head, lateral view **14** propodeum and TI-III, dorsal view. Scale line = 0.5 mm.

### Genus Wilkinsonellus

Mason, 1981

Wilkinsonellus Mason 1981, 115: 122; Austin and Dangerfield 1992, 6(1): 23; Chen and Song 2004: 202; Whitefield 1997, 1: 12. **Type species**: *Apanteles iphitus* Nixon, 1965. Designated by Mason 1981.

#### Diagonosis.

Areolet of fore wing absent; nervellus of hind wing not sinuate. Propodeum often with a medial longitudinal carina, sometimes smooth or rugulose or strigose with indistinct carina. TI-TIII not forming a carapace; TI 4–5 times as long as its apical width, more or less constricted medially and deeply grooved almost to apex.

### Wilkinsonellus paramplus

Long & van Achterberg, 2003

http://species-id.net/wiki/Wilkinsonellus_paramplus

Figs 15–20

Wilkinsonellus paramplus Long& van Achterberg, 2003, 77(10): 223. Holotype female, pinned with labels as follows: “[Vietnam], Hoa Binh (Yen Thuy), 20°23'N, 105°36'E, fruit orchard, MT, 20–30.v.2002, K.D. Long, Mic. 243” (IEBR).

#### Materials examined.

1♀, Mt. Nanling (112°59'09"E, 24°53'46"N ~ 113°05'28"E, 24°56'42"N), Ruyuan, Guangdong, 2003. VII. 23, Xu Zaifu, No. 20049062; 1♀, Shiwandashan Forest Park(107°53'E, 21°53'N ~ 107°55'E, 24°55'N, 310m), Guangxi, 2001. XI. 29, Ma Yun, No. 20021580.

#### Distribution.

China (Guangdong, Guangxi), Vietnam. **New record for China.**

**Figures 15–20.**
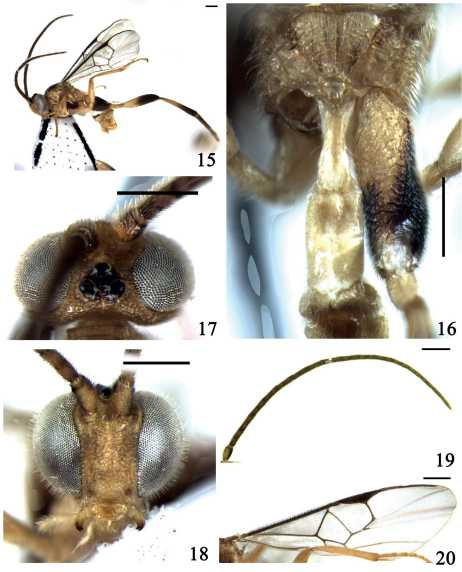
*Wilkinsonellus paramplus* Long & van Achterberg, 2003 **15** habitus, lateral view **16** propodeum and anterior tergites of gaster, dorsal view **17** head, dorsal view **18** head, frontal view **19** antennae **20** fore wing. Scale line = 0.5 mm.

## Supplementary Material

XML Treatment for Deuterixys

XML Treatment for Deuterixys bifossalis

XML Treatment for Deuterixys curticalcar

XML Treatment for Wilkinsonellus

XML Treatment for Wilkinsonellus paramplus
